# Rapid Generation of Monoclonal Antibodies from Single B Cells by Ecobody Technology

**DOI:** 10.3390/antib7040038

**Published:** 2018-11-07

**Authors:** Teruyo Ojima-Kato, Shiomi Morishita, Yoshino Uchida, Satomi Nagai, Takaaki Kojima, Hideo Nakano

**Affiliations:** 1 iBody Inc., Furo-cho 1, Chikusa-ku, Nagoya 464-0814, Japan; nagai.satomi@molbiotech-nagoya.org (S.N.); hnakano@agr.nagoya-u.ac.jp (H.N.); 2 Graduate School of Bioagricultural Sciences, Nagoya University, Furo-cho, Chikusa-ku, Nagoya 464-8601, Japan; morishita.shiomi@molbiotech-nagoya.org (S.M.); uchida.yoshino@molbiotech-nagoya.org (Y.U.); kojimat@nuagr1.agr.nagoya-u.ac.jp (T.K.)

**Keywords:** single B cell technology, rabbit antibodies, human antibodies

## Abstract

Single B cell sampling following to direct gene amplification and transient expression in animal cells has been recognized as powerful monoclonal antibodies (mAbs) screening strategies. Here we report Ecobody technology which allows mAbs screening from single B cells in two days This technology uses *Escherichia coli* cell-free protein synthesis (CFPS) for mAb expression. In the CFPS step, we employed our original techniques: (1) ‘Zipbody’ as a modified Fab (fragment of antigen binding) format, in which the active Fab formation is facilitated by adhesive leucine zipper peptides fused at the C-termini of the light and heavy chains; and (2) an N-terminal SKIK peptide tag that can markedly increase protein production. By the Ecobody technology, we demonstrated rapid screening of antigen specific mAbs from immunized rabbits and Epstein-Barr Virus infected human B cells. We further obtained rabbit mAbs in *E. coli* expression system yielding to 8.5 mg of purified proteins from 1 L bacterial culture.

## 1. Introduction

Monoclonal antibodies (mAbs) have become essential agents for research, diagnostic and therapeutic areas.

Single B cell screening technologies, which can rapidly generate mAbs from sampled single B cells from immunized animals, have been proven to be powerful techniques to obtain the natural antibody repertoire [1,2,3,4,5]. Usually in these methods, recombinant production of the mAbs is performed using animal cells like CHO and HEK293, resulting in a rate-limitation of the screening process, because transfection and expression in animal cells requires at least 3–5 days. In contrast, cell-free protein synthesis (CFPS) offers an alternative expression system that avoids many of the problems of conventional cell-based expression technologies [6,7]. CFPS has big advantages over in vivo methods for high-throughput recombinant protein production without requiring time-consuming gene-cloning, transformation, or cultivation [8].

Taking advantage of CFPS systems, we developed a rapid mAb screening system named “Single-Cell Reverse Transcription-PCR linked in vitro Expression (SICREX)”, which utilizes *Escherichia coli* extract-based CFPS systems to produce fragments of antigen binding (Fab) derived from single B cells [9,10,11]. Using this method, proteins can be rapidly synthesized just by mixing the *E. coli* cell-extract with PCR-amplified DNA templates, amino acids, nucleotides, T7 RNA polymerase and an energy source. However, practically available mAb screening was challenging due to the following technical problems. Firstly, active Fabs were sometimes not formed in the CFPS because of incorrect folding and assembling of heavy chain (Hc) and light chains (Lc). In particular, active Fabs were not produced at all in the case of rabbit mAb clones, probably because of the presence of too many Cys residues involved in disulfide bond formation [11,12]. Therefore, reconstruction of single chain Fv (scFv) genes was required for enzyme-linked immunosorbent assay (ELISA) evaluation. Secondly, the protein production levels significantly depend on the clones or genes and it was difficult to obtain enough mAb proteins in CFPS for ELISA evaluation. In some cases, the amount of Hc and Lc gene templates included in the CFPS should be optimized [13,14]. To overcome such limitations, we have recently developed a modified Fab format named ‘Zipbody’ that contains adhesive short peptides leucine zippers (LZ) at the C-terminus of the Hc and Lc, respectively. We found that the fusion of the LZ to the Fab could enhance correct pairing of the Hc and Lc, leading to the formation of active Fab in both *Escherichia coli* CFPS and living cell expression systems [15]. Furthermore, we found that the protein production levels can be markedly improved by just inserting 12 nucleotides next to the start codon [16]. This sequence encodes a short peptide Ser-Lys-Ile-Lys (SKIK).

Together with Zipbody and the SKIK peptide tag technologies, for improvement of Fab formation and protein production in CFPS, we have developed an improved SICREX system renewed as ‘Ecobody technology’ [17]. Here, we demonstrate a 2-day protocol to complete screening of antigen-specific mAbs from single B cells of rabbits and Epstein-Barr Virus (EBV) infected human B cells. We further describe active Zipbody production in *E. coli* cytoplasmic expression system followed by refolding of inclusion bodies.

Ecobody technology will be beneficial to the field of mAb research and development as a high-throughput and low-cost mAb screening method.

## 2. Materials and Methods

### 2.1. Overview of Ecobody Technology

**Figure 1:**
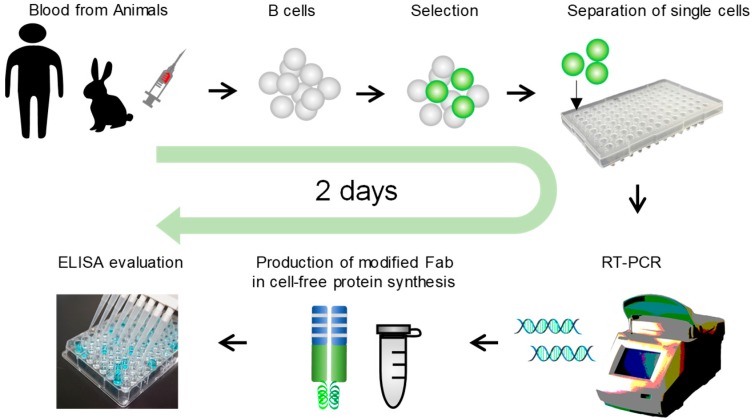
Scheme of the Ecobody technology. The whole process is seamlessly carried out in vitro. The modified Fab (fragment of antigen binding) format ‘Zipbody’ and ‘N-terminal SKIK peptide tag’ are the key techniques to obtain enough and active monoclonal antibodies (mAb) proteins in *Escherichia coli* cell-free protein synthesis.

The scheme of Ecobody technology is illustrated in Figure 1. The details are designed as below; (i) Collect blood samples from immunized animals or human donors. (ii) Collect lymphocytes by density gradient centrifugation. (iii) Select target B cells by such as fluorescent reagents and magnetic beads. (iv) Separate single cells per wells by fluorescence-activated cell sorting, limiting dilution method, or some other devices like micromanipulator. (v) RT-PCR from single B cells to prepare Zipbody genes fused with N-terminal SKIK peptide tag. This step includes cell direct reverse transcription with mAb genes specific primers (15 min), first PCR to amplify Hc and Lc (1 h), second PCR to connect the required DNA tails for the following DNA assembly (1 h), Gibson assemble with the vector which contains sequences of T7 promoter, N-terminal SKIK peptide tag, Zipbody construct, His tag or HA tag, and T7 terminator (15 min), and final PCR to prepare Hc and Lc DNA fragments for expression. (vi) *E. coli* based cell-free protein synthesis (1.5 h). (vii) mAbs evaluation by ELISA (3 h).

### 2.2. Preparation of Antigens and Immunization of Rabbits

Three types of antigens, bacteria *Vibrio parahaemolyticus* NBRC 12711, *E. coli* O26 GTC14538 (verotoxin-1 producing strain), and non-toxic verotoxin 2 (VT2) were used as antigens for immunization of rabbits. Bacteria were obtained from the Biological Resource Center at the National Institute of Technology and Evaluation (NITE, Kisarazu, Japan) and the National BioResource Project GTC Collection (Gifu, Japan), cultured in Luria-Bertani (LB) medium at 37 °C overnight, and inactivated by incubating at 80 °C for 30 min in phosphate-buffered saline (PBS) containing 0.25% formalin. They were stored at −20 °C. Nontoxic VT2 (E167Q mutant) expression vector was prepared by PCR from VT2 producing *E. coli* O157 GTC 14535 strain with the primers AAGAAGGAGATATACATATGAAGTGTATATTATTTAAATGGGTACTG (forward) and TGGTGGTGGTGGTGCTCGAGGTCATTATTAAACTGCACTTCAGCAAAT (reverse) using KOD FX polymerase (Toyobo, Osaka, Japan). After NdeI and XhoI treated PCR product and pET22b vector were ligated, inverse PCR introducing E167Q mutation into VT2a subunit was performed with primers ACAGCACAAGCCTTACGCTTCAG and TAAGGCTTGTGCTGTGACAGTG using KOD Plus polymerase (Toyobo). The His-tagged nontoxic VT2 was expressed in BL21 (DE3) pLysS and purified using Ni-Sepharose 6 Fast Flow resin (GE healthcare, Little Chalfont, UK) and HT-Hydroxy apatite (Bio-rad, Hercules, CA, USA) as described by He et al. [18]. The two bands of subunit VT2a E167Q (36 kDa) and 2b-His tag (11 kDa) were confirmed by CBB staining after SDS-PAGE. New Zealand White rabbits (2–3 weeks) were immunized with 10^8^ dead bacterial cells or 0.2 mg/mL of nontoxic VT2 supplemented with 2.5 mL of complete Freund’s adjuvant by hypodermic injection. The second and third boosters were given after intervals of 2 weeks and 10 days, respectively. A few mL of blood samples was collected 2 days after the final booster. This study was approved by the Committee of Animal Experiments of the Graduate School of Bioagricultural Sciences, Nagoya University (permit number 2015022611) and performed according to the Regulations on Animal Experiments in Nagoya University.

### 2.3. Preparation of EBV Infected Human Cells

The EBV infected cell line HEV0011, provided by the RIKEN BRC (Tsukuba, Japan) through the National Bio-Resource Project of the MEXT/AMED [19], was once cultured in RPMI1640 medium with 20% FBS in T75 flask at 37 °C with 5% CO_2_. The proliferated cells (10^6^ cells/tube) were stored in 1 mL of the same medium supplemented with 10% dimethyl sulfoxide in liquid nitrogen.

### 2.4. Selection of B Cells

#### 2.4.1. Selection of Rabbit B Cells Binding to Bacterial Cells

After a few mL of blood samples were collected from the immunized rabbits, lymphocytes were separated by a previously reported protocol using density-gradient centrifugation [11]. To select matured B cells, 10^6^ cells/mL-PBS were stained with 1 µM ER-Tracker™ Green (BODIPY^®^ FL Glibenclamide, Life Technologies, Foster City, CA, USA) at room temperature for 5 min then centrifuged at 1000× *g* for 2 min as described by Kurosawa et al. [2]. The cells with higher fluorescence intensity were then sorted by fluorescence activated cell sorting (FACS, JSAN; Bay Bioscience, Kobe, Japan). Next, the sorted cells were selected with antigen-coated magnetic beads as described previously [11]. In brief, biotinylated dead bacterial cells conjugated with DynaBeads M-280 Streptavidin (SA beads, Life Technologies) were prepared and these beads were used to concentrate B cells that bound to the antigens after removing cells non-specifically bound to SA-beads and *E. coli* DH5alpha-coated beads. After that, the remaining B cells were separated into each well (1 cell/10 µL-PBS) of 0.2 mm glass-bottomed 384-well imaging plates (Corning, Corning, NY, USA) and the bead-B cell complexes in each well were confirmed under a phase-contrast inverted microscope (CKX53; Olympus, Tokyo, Japan). Bürker-Türk plates were used for cell counting.

#### 2.4.2. Selection of Rabbit B Cells Producing mAbs to Nontoxic VT2

The lymphocytes fraction separated by the same method as above was stained with ER-tracker and HiLyte Fluor 647 labeled nontoxic VT2 which was prepared by HiLyte Fluor^TM^ 647 Labeling Kit-NH_2_ (Dojindo Laboratories, Kumamoto, Japan). After replacing the solution with new PBS, the cells with double positive signals were separated into a 96 well PCR plate containing 10 µL of RT reaction mixture/well by the single cell sorting mode of cell sorter SH800 (SONY, Tokyo, Japan).

#### 2.4.3. Selection of Influenza Vaccine Reactive EBV Infected Human Cells

The stored HEV0011 cells were sub-cultured in a T25 culture flask overnight. Then, the cells were incubated with 11 µL/mL of HiLyte Fluor 647 labeled anti-Human IgG (Fc specific) antibody produced in goat (Merck, Kenilworth, NJ, USA) in PBS at 20 °C for 20 min. The cells were centrifuged (1000× *g* for 3 min) to replace the solution with 1 mL new PBS. The cells with higher HiLyte Fluor 647 fluorescence were collected by FACS (JSAN Bay Bioscience, Kobe, Japan). Next, the sorted cells (10^3^ cells/100 µL PBS) were mixed with 5 µL of magnetic beads coated with *E. coli* DH5alpha (6 × 10^5^ beads/µL PBS) and incubated at room temperature for 10 min. After removing beads fraction with a magnet stand, cells in the supernatant were mixed with 5 µL of magnetic beads coated with influenza vaccine (Biken HA, manufactured in 2017, containing A/Singapore/GP1908/2015 (H1N1) pdm09, A/Hong Kong/4801/2014 (H3N2), B/Phuket/3073/2013 (Yamagata strain), and B/Texas/2/2013 (Victoria strain)) (6 × 10^5^ beads/µL PBS) and incubated as well. The cells bound to the beads were collected, diluted to 1 cell/10 µL PBS, and separated into a 384 well glass plate to be 1 cell/well.

### 2.5. RT-PCR

Reverse transcription from the single cell, 1st PCR, and 2nd PCR were serially carried out as described previously [17]. In the case of human mAbs, variable regions of kappa and lambda types of Lc, and gamma and mu of Hc were separately amplified. The 2nd PCR products were assembled with the linearized pRSET-based cloning vectors containing T7 promoter, N-terminal SKIK peptide tag, constant regions of Lc or Hc, LZ (LZA or LZB), His or HA tag, and T7 terminator.

### 2.6. Cell-Free Protein Synthesis (CFPS)

To express SKIK-fused Zipbody, the DNA templates of Hc and Lc were amplified from the above assembled vectors with primers annealed to upstream of the T7 promoter and downstream of the T7 terminator. *E. coli*-based PURE system (GeneFrontier, Kashiwa, Japan) with oxidized glutathione (GSSG) and DsbC were used as recommended by the manufacturer. Proteins were expressed on a 10-µL scale containing 0.5 µL PCR product or 20 ng purified DNA fragments (each of Hc and Lc) as the template, at 37 °C for 90 min. FluoroTect™ Green_Lys_ in vitro Translation Labeling System (Promega, Madison, WI, USA) was included to confirm protein synthesis in subsequent SDS-PAGE analysis.

### 2.7. ELISA

Unless otherwise stated, mAbs produced in CFPS or *E. coli* were analyzed by ELISA using our standard protocol described previously. In brief, 50 µL of dead bacterial cells (OD600 nm = 0.1) or 10 µg/mL protein antigens (nontoxic VT2 or influenza vaccine), 0.4% Bovine Serum Albumin (BSA) in PBS as negative control were coated on the MaxiSorp plate (Thermo) overnight, and we followed the standard ELISA protocol using Zipbody as the primary antibody, and anti-rabbit Ig(G + M)-HRP conjugate (Southern Biotech, Birmingham, AL, USA), anti-His tag-HRP conjugate as the secondary antibody, or anti human IgG(H + L) polyclonal antibody-HRP conjugate produced in goat (GeneTex, Irvine, CA, USA). Can Get Signal Immunoreaction Enhancer Solution 2 (Toyobo) was used for dilution of the secondary antibody.

### 2.8. mAb Production in E. coli

To produce the Zipbody with an SKIK tag, Hc and Lc were coexpressed in *E. coli* Shuffle T7 Express (New England Biolabs, Ipswich, MA, USA) as the host, grown in 50 mL LB medium supplemented with 100 µg/mL ampicillin with 1 mM IPTG induction at 16 °C for 24 h, as described in our previous study [17]. The inclusion bodies were refolded by the stepwise dialysis method using guanidinium hydrochloride [20]. In the case of anti-nontoxic VT2 mAb clone, Hc and Lc genes independently cloned into pET22b were separately expressed in *E. coli* BL21star (DE3) hovering plasmid pSJS1240 in the same medium with 100 µg/mL spectinomycin at 37 °C for 4.5 h. The amount of inclusion bodies of Hc and Lc was equalized and refolded together in the single dialysis tube. The KD values were measured by Bio-layer interferometry method.

## 3. Results

### 3.1. Rabbit mAbs Screening

We followed the workflow of Ecobody technology shown in Figure 1 to develop mAbs that bind to *V. parahaemolyticus*, *E. coli* O26, and nontoxic VT2. The details are described in the following sections.

#### 3.1.1. *V. parahaemolyticus*

**Figure 2:**
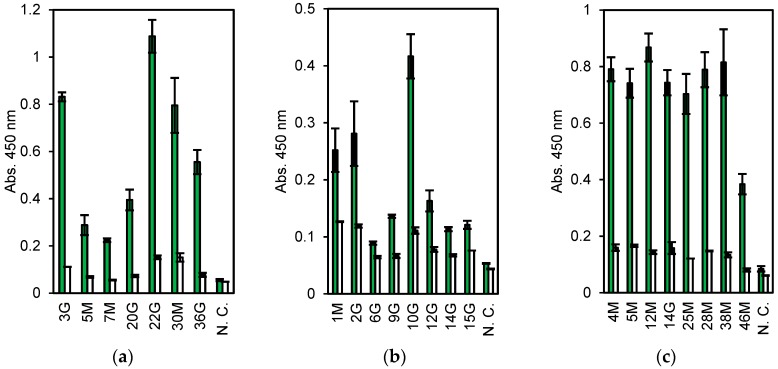
Enzyme-linked immunosorbent assay (ELISA) results of the cell-free products. Antigens are (**a**) *Vibrio parahaemolyticus*. (**b**) *Escherichia coli* O26, and (**c**) nontoxic VT2. Green: Antigen, White: Bovine serum albumin (BSA). N. C. means no DNA templates in the cell-free protein synthesis (CFPS) reaction. Error bars indicate standard deviations.

First, 6.8 × 10^6^ lymphocytes were collected from a few milliliters of blood of a rabbit immunized with dead *V. parahaemolyticus*. The cells, about 30% (2.0 × 10^4^ cells), showing higher fluorescence stained with ER tracker were collected by FACS. Subsequently, approximately 500 cells bound to magnetic beads coated with dead *V. parahaemolyticus* were separated by magnet, of which 19 were used for single-cell RT-PCR. From them, seven pairs of Lc and Hc genes were obtained (three IgM and four IgG clones, named 3 G, 5 M, 7 M, 20 G, 22 G, 30 M, and 36 G). They were expressed in CFPS and evaluated by ELISA. All the clones, expressed as the Zipbody with the N-terminal SKIK tag, had higher binding activity toward *V. parahaemolyticus* than toward the other tested antigens (Figure 2a). Clone 22 G had the highest ELISA signal and low cross-reactivity.

#### 3.1.2. *E. coli* O26

The mAb screening process for anti-*E. coli* O26 was almost the same as that described above, except for an additional step to remove B cells cross-reacting with *E. coli* O111 before selection with *E. coli* O26-coated beads. From 3.0 × 10^6^ lymphocytes, 5.4 × 10^4^ cells were sorted by FACS and finally 750 cells were collected with *E. coli* O26-coated beads. From these, 22 cells were subjected to single-cell PCR and 10 pairs of Hc and Lc genes (clone names 1 M, 2 G, 3 M, 5 G, 6G, 9 G, 10 G, 12 G, 14 G, and 15 G) were obtained (data not shown). As the DNA of 3 M and 5 G were not amplified by PCR following Gibson assembly, in total, eight clones were expressed in CFPS and evaluated by ELISA, and each showed a higher signal with *E. coli* O26 than with BSA (negative control) (Figure 2b).

#### 3.1.3. Nontoxic VT2

For nontoxic VT2 mAbs, 6.2 × 10^5^ cells were used and 165 cells (0.26%) were selected as ER tracker positive and antigen specific ones. From these, 47 cells were sorted into PCR tubes with single cell sorting mode. Of which, 8 pairs of Hc and Lc genes (clone names 4 M, 5 M, 12 M, 14 G, 25 M, 28 M, 38 M, and 46 M) were finally obtained and subjected to CFPS and ELISA. All clones showed high ELISA signals toward antigen (Figure 2c).

### 3.2. Expression in E. coli

**Figure 3:**
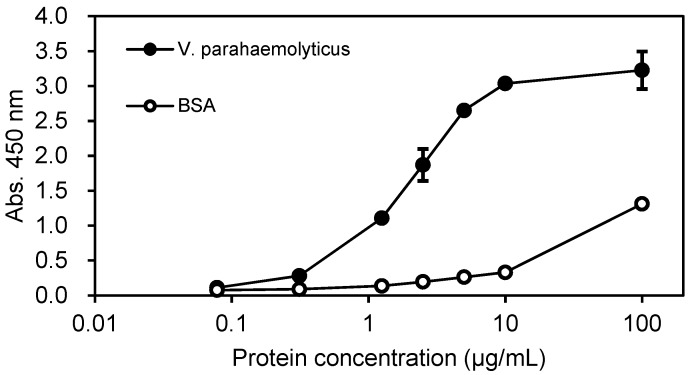
ELISA results of the refolded and purified Zipbody 22 G clone for *V. parahaemolyticus.* Error bars indicate standard deviations.

**Figure 4:**
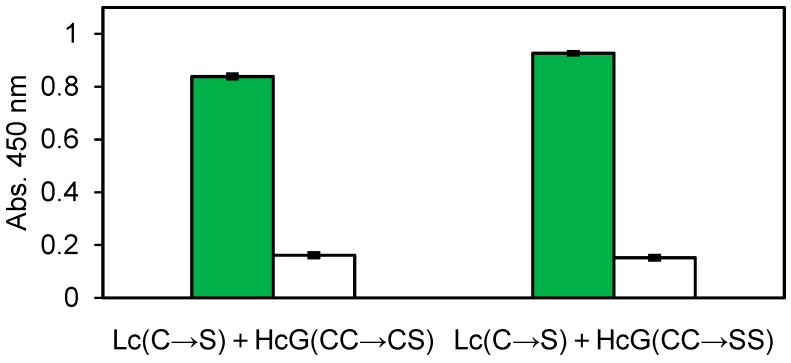
ELISA result of the refolded 14 G mutated clones for nontoxic VT2. Green: Antigen, White: BSA. Error bars indicate standard deviations.

From the above obtained mAb clones, 22 G for *V. parahaemolyticus* and 14 G for nontoxic VT2 were selected for next *E. coli* expression and further investigation. The Zipbody with N-terminal SKIK peptide tag of clone 22 G was expressed in *E. coli* Shuffle T7 Express. Since most of the target proteins were produced as inclusion bodies, refolding was tried. As a result, 82.8% of the refolded proteins was recovered as soluble fraction (Table 1). Finally, 27.4% was recovered as purified protein by His tag purification. Using this purified Zipbody as the first antibody, binding activity for the antigen was observed in ELISA (Figure 3). In the case of 14 G clone for nontoxic VT2, Cys residues in constant regions which might be involved in intermolecular disulfide bonds were converted to Ser. In this study we constructed two mutants, (1) Lc (C→S), Hc (CC→SC) and (2) Lc (C→S), Hc (CC→SS), then Lc and Hc were independently expressed in BL21 (DE3). As this also resulted in protein production in inclusion bodies, Lc and Hc were mixed and refolded as described above. ELISA showed enough binding activity to the antigen (Figure 4), implying expression in BL21 (DE3) and refolding the mixture of Lc and Hc would be effective for active rabbit mAb production.

Inclusion bodies produced in 50 mL of LB medium were used. The solubilized amount of protein is regarded as 100% in recovery ratio.

### 3.3. Human mAbs Screening

**Figure 5:**
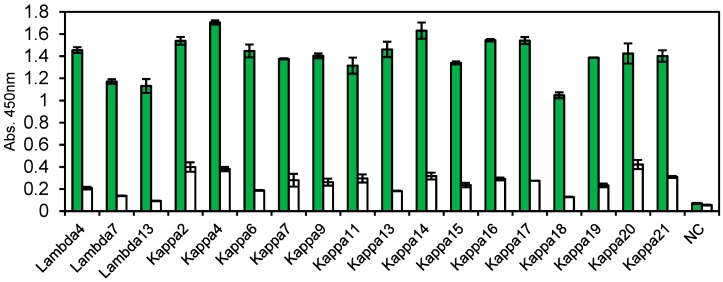
ELISA results of the cell-free products obtained from human B cells. Green: Influenza vaccine, White: BSA. Error bars indicate standard deviations.

We attempted to obtain anti-influenza virus vaccine human mAbs from immortalized B cells by Ecobody technology. In order to select IgG-producing B cells, anti-human IgG antibody labeled with a fluorescent dye was used and B cells with highest fluorescence (about 30%) were sorted by FACS. After removing cells nonspecifically bound to *E. coli* DH5α dead cells, approximately 0.15% of the starting cells were collected by antigen coated magnetic beads. Of which, 21 cells were selected under microscope (Figure 5) and subjected to single-cell RT-PCR. Among these 15 pairs of Lc (kappa type) and Hc (gamma type) genes were obtained. Lambda type of Lc was also amplified from three cells. Therefore, a total of eighteen pairs of Lc and Hc were coexpressed in CFPS and 6-times diluted products were evaluated by ELISA. Figure 5 shows that all clones obtained in this screening, regardless of the types of Lc, had higher binding activity to the antigen.

**Figure 6:**
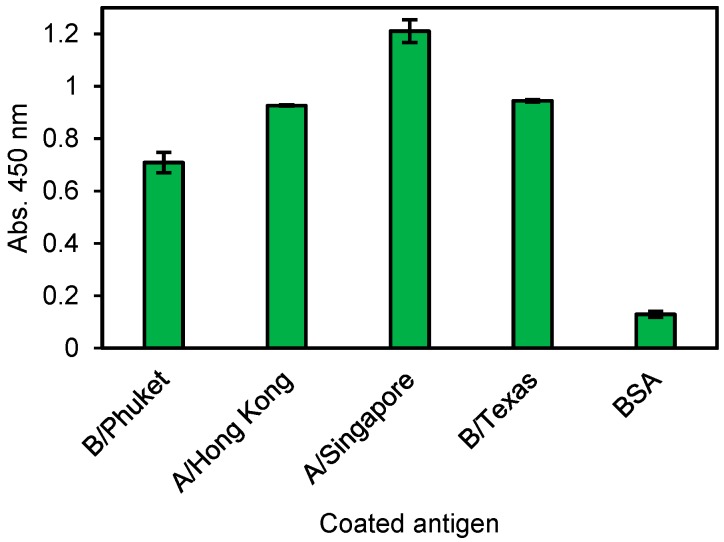
ELISA result of the *E. coli* expressed and refolded human No. 14 G Zipbody. Each antigen was coated on ELISA plate and 10 ng/µL of His tag purified Zipbody was used as the primary antibody. Error bars indicate standard deviations.

No. 14 Zipbody was expressed in *E. coli* Shuffle T7 Express. Although most of the target proteins were confirmed in the insoluble fraction, they were also in the soluble fraction (data not shown). Therefore, we tried His tag purification from the soluble proteins and ELISA was conducted against individual influenza antigen, not vaccine mixture. As a result, this clone had reactivity to all (Figure 6). From this result, it was considered that No. 14 clone could recognize the common site of each influenza HA antigen.

## 4. Discussion

Rapid discovery of mAbs which have high specificity and affinity to the antigens is important for accelerating life science research and industry. Here, we reported “Ecobody technology”, allowing rapid screening of mAbs from single B cells of animals.

Using the immunized rabbits and immortalized human B cells, we demonstrated that it was possible to complete the process from isolation of B cells to mAb evaluation by manual operation in just two normal working days. The obtained mAb clones were produced as Zipbody proteins with an SKIK tag in CFPS and had binding activity to relevant antigens (Figure 2 and Figure 5). All of the clones had binding activities to the target antigens. While pathogenic bacteria and protein were used as the antigen in this work, a rapid mAb screening by Ecobody technology will be available for challenging antigens like small chemicals and membrane proteins because B cells expressing mAbs against such antigens can be isolated by the same strategy with magnetic beads or FACS. In addition, as all the processes are carried out in tubes with only simple pipetting operations, Ecobody technology would be automated. This is a distinct advantage over other single B cell methods using animal cells, in which transfection and culturing of cells are necessary. We believe a rapid mAb screening would have great benefit for accelerating the mAb industry. In this study, most of the processes were performed with manual operations, and single B cell selection using microscopy was laborious, but the system could become more efficient by replacing manual operations to machine mediated processes.

Two rabbit mAb clones (22 G for *V. parahaemolyticus* and 14 G for nontoxic VT2) and human mAb (No. 14 for influenza virus vaccine) with high ELISA signals were expressed in *E. coli.* Consistent with our previous study about the SKIK peptide tag [16], most of the target proteins were produced in insoluble fraction (data not shown). Therefore, we tried refolding the insoluble Zipbody by the method reported for scFv, and successfully recovered >80% of the insoluble proteins in a soluble form without optimization of the refolding process. His-tag purification of the refolded protein yielded about 8.5 mg of purified 22 G Zipbody from 1 L of LB medium (Table 1). The refolded and purified Zipbody had Hc-Lc association and the binding activity was sufficiently high (KD = 469 pM, data not shown) when compared with that of natural rabbit mAbs, which have KD values of a few hundred pM [17]. Interestingly two mutants in which particular Cys residues were removed also showed the ELISA signals after refolding (Figure 4), indicating Lc and Hc might be associated with LZ interaction. The molecular structure and stability of the refolded mAbs, and the versatility of this method to obtain other mAb clones must be investigated, but we expect that rapid screening of mAbs by Ecobody technology coupled with *E. coli* expression has the potential to provide low cost mAb reagents applicable for diagnostics or research uses.

In conclusion, we adopted two techniques, Zipbody and the N-terminal SKIK peptide tag, and succeeded in developing a mAb screening system, Ecobody technology, allowing rapid generation of mAbs derived from single B cells.

## 5. Conclusions

Ecobody technology will be beneficial for rapid rabbit and human mAb screening.

## Figures and Tables

**Table 1:** Recovery of refolding and purification of 22 G clone expressed in *E. coli* Shuffle T7 Express.

	Protein Concentration (mg/mL)	Total Volume (mL)	Amount of Protein (mg)	Recovery Ratio (%)
Solubilization by 6 M GuHCl	1.62	0.95	1.54	
Refolding	0.98	1.3	1.28	82.8
His tag purification	0.094	4.5	0.423	27.4
